# Identifying the key features and outcomes of family navigation services for mental health and/or addictions concerns: a Delphi study

**DOI:** 10.1186/s12913-019-3968-6

**Published:** 2019-02-28

**Authors:** Roula Markoulakis, Samantha Chan, Anthony Levitt

**Affiliations:** 1 Sunnybrook Research Institute, Toronto, ON Canada; 2 Sunnybrook Health Sciences Centre, Toronto, Canada

**Keywords:** Youth, Families, Mental health, Addictions, Delphi method, Navigation

## Abstract

**Background:**

Family navigation in mental health and addictions is a mode of support aimed at helping families through the complex mental health and addictions system, making well-informed service matches, and engaging with families throughout their care journeys. As family navigation services emerge and grow, understanding their unique features and impacts is essential to defining evaluation measures and driving good outcomes for families.

**Methods:**

This Delphi study investigated the defining features of family mental health and addictions navigation, factors involved in a successful service match, and important outcomes of the process through perspectives of clients and team members of a family navigation program, as well as those of local mental health and/or addictions service providers. In the first phase, participants (*n* = 41), were asked to respond to a series of prompts pertaining to 1) the key features of a successful family navigation process, 2) the features of good matches between youth or families and the services to which they are navigated, and 3) the outcomes of importance in family navigation. In Phase 2, findings from Phase 1 were presented to participants (*n* = 32) to select and rank their top ten responses to each prompt. Responses which passed a cut-point were carried into Phase 3, in which participants (*n* = 20), rated the importance of the remaining items. Items rated as “very” or “extremely” important by 80% or more of participants in Phase 3 had achieved consensus. Intra-class correlation coefficients were calculated to confirm participant agreement on all items having achieved consensus.

**Results:**

Sample items with 100% consensus were as follows: navigator determines the best fit by understanding and considering the youth and families’ needs, by collaborating with team members and service providers, and by providing individualized suggestions; navigation involves knowledge and understanding of mental health and addictions system and existing services; referred service providers are knowledgeable and up-to-date on evidence-based practice and have multidisciplinary perspectives in service. Overall ICC across all finalized statements following Phase 3 was .84.

**Conclusions:**

Exploring the key features of successful navigation, outcomes of importance to stakeholders, and elements of successful matches can inform the development of navigation services that address families’ needs, can support service providers in ensuring well-matched services, and lend vital support to families seeking services within a complex system.

**Electronic supplementary material:**

The online version of this article (10.1186/s12913-019-3968-6) contains supplementary material, which is available to authorized users.

## Background

People with mental health and/or additions (MHA) issues experience physical, emotional, and social strains as a result of their illnesses, while also trying to find their way through an often unfamiliar and frequently fragmented healthcare system. Navigation, which initially emerged as a care strategy for breast cancer patients to address financial, communication, information, systemic, and emotional barriers that could arise in the complex and fragmented care system in place for this illness [1] has recently been adapted for people needing to access support in the mental health and/or addictions care system. MHA navigation programs aim to help patients by reducing barriers, connecting them to appropriate resources and supports in a timely manner, and empowering patients in managing their health [2–4].

Navigation can be particularly suitable for youth with MHA concerns and their families. MHA issues tend to first appear in adolescence and early adulthood, making early intervention critical in reducing the later burden of illness for the youth and family [5]. When youth do not receive appropriate support, they may disengage from care and be placed at increased risk of experiencing lasting MHA concerns and associated consequences [6]. Youth MHA issues can place increased demand on caregivers, leading to impacts on family health and productivity [5, 7]. Although families are often intimately involved in the MHA care of a youth, their needs are often overlooked or unsupported within the existing mechanisms of youth MHA care. Support for access to care is thus essential for a youth and should also include support for the whole family. Navigation services can work to connect youth to the most appropriate services for their needs, while taking a family-focused approach that supports family members and also connects them to services and resources as necessary.

As a result of the vital role families play in ensuring health care delivery and the potential negative impact of youth illness on the whole family, services that support families of youth with mental health and addictions service needs in navigating the system have recently seen increased implementation, for example, across Canada [4], the United States [8], and the United Kingdom [9]. In general, family navigation services support youth with MHA issues and their families in connecting with needed services [4]. An environmental scan of pediatric navigation models in Canada identified that many existing navigation programs have similar goals, including advocating for and educating patients, as well as supporting and assisting them in accessing services within and outside the healthcare system [10]. Given the newness of such services, there is limited understanding of the key features of navigation for this group, as well as the outcomes of importance to those involved in navigation processes. A review of literature pertaining to MHA navigation services (Mullen, unpublished data) identified three core components of these services: client-centered support, barrier reduction, and integrated care. However, it was unclear from the available literature what features of navigation processes contribute to these core components and whether these also specifically pertain to family-focused navigation services (henceforth referred to as family navigation). Furthermore, the few studies that have explored the outcomes of MHA navigation suggest that navigation is associated with a reduction in barriers to health care and substance abuse services [3], improved access to MHA care providers [11], decreases in current health problems, and in visits to primary care providers [12]. Although these early findings are promising, outcomes of importance in navigation have not been clearly articulated, thereby limiting understanding of the potential value of navigation or consistent exploration of outcomes across programs and studies. Thus, there are few sources reporting on outcomes of MHA navigation and a lack of consistency in the outcomes selected, without indication of meaningfulness to families, service providers, or navigation programs.

Identification and measurement of outcomes in family navigation are also unique challenges. Firstly, such challenges arise due to the complexity involved in tracking outcomes for patients (e.g., youth in particular, and on occasion, family members in need of support) who are navigated to receive services elsewhere, meaning the navigation service does not deliver the actual treatment. Secondly, further challenges arise due to the difficulties associated with obtaining such information when a family member may be the Navigator’s primary point of contact rather than the eventual service recipient (i.e., the youth is the treatment or service recipient, but the family member may be the primary contact person for the navigation program). Overall, understanding outcomes of importance in family navigation will become increasingly critical as these services are implemented as a support for complex youth mental health and addictions issues. Identifying priorities and defining success for family navigation services can help create a unified understanding of this innovative service model for families and guide directions in the delivery of care, and assist in consistency of outcome assessment across research in the field. Thus, the purpose of this study was to describe the core functions of family navigation, namely by obtaining consensus regarding: 1) the key features of a successful family navigation process, 2) the features of good matches between youth/families and the services to which they are navigated, and 3) the outcomes of importance in family navigation.

## Methods

### Delphi method

This study sought input from a range of content experts in order to reach consensus on the key features of a successful navigation process for youth with mental health and/or addictions concerns and their families, the features of a good “match” for youth, families, and service providers, and outcomes of importance in navigation. The Delphi method is appropriate for collecting informed judgments across a range of disciplines and attains consensus from a group of experts on a specific topic [13, 14]. Through multiple iterations of questionnaires, identified experts are given the opportunity to provide their opinions on a topic of interest and consider their opinions in light of group opinions. Furthermore, a web-based Delphi approach can create a forum for participants to voice their perspectives anonymously, thereby preventing confrontation or pressure to conform to dominant group ideas, as would be possible in face-to-face focus group settings [14, 15]. For instance, in the current study there was concern that clients with lived experience would feel unnecessary pressure to conform to the views expressed by service providers, who they might view as experts in the MHA system, thereby undervaluing their own expertise in this area. Furthermore, due to its asynchronous implementation, time and geographical barriers can be mitigated via this method, which helps encourage participation from those who would otherwise be unable or unlikely to meet in person [15], such as individuals caring for a youth who is unwell or service providers who would be unable to leave their own practice during the day to participate in the research. Although this method requires a disproportionately large time investment from researchers due to time between individual responses and between study phases, it makes feasible the collection of data that represents a wide range of expert opinions on the topic of interest [14]. We therefore employed this web-based, asynchronous Delphi process for the current study.

### Setting

To effectively explore and describe the core components of family navigation in mental health and addictions, one program in particular was the primary focus in order to ensure depth of conceptual development. The FNP at Sunnybrook Health Sciences Centre is a program for families of youth ages 13–26 with mental health and/or addictions concerns that provides expert navigation from a clinically-trained health professional in order to match youth and families with the most appropriate services and supports to enhance their health and functioning [4]. The FNP is the largest such program in Canada and serves a large number (approximately 750) of clients/families annually. The Family Advisory Council consists of family members with lived experience of caring for youth with MHA issues, and oversees the activities of the (FNP).

### Participants

Participants in traditional Delphi studies are often trained experts in the area of interest. The expert panels in this study comprised three groups. The first was Family Navigation Project (FNP) team members, that included Navigators and other clinical staff, management, and members of the FNP Family Advisory Council. The second group comprised mental health and/or addictions service providers within the Greater Toronto Area who receive referrals from or make referrals to the FNP, thus lending knowledge of navigation from the lens of the broader MHA system. The third group was composed of current and/or former clients of the FNP, which included parents/caregivers of youth with mental health and/or addictions concerns and youth with lived experience of mental health and/or addictions concerns. This third expert panel was included to reflect the expertise embodied in lived experience, which has been a key principle driving program development at FNP since its inception, and with the additional perspective of having received family navigation services. Although parents and youth might not have formal training in the mental health field, they are considered to have “expert knowledge” with regard to their own experiences of mental health and/or addictions issues, navigating through the complex mental health and addiction systems on their own and with the support of FNP, and seeking appropriate service matches. Although “experts” are generally considered to be those with professional background in the area of interest in Delphi studies, it was considered relevant and important to this research to ensure that individuals with lived experience were seen as experts and given equal voice with professionals in the MHA system [16]. The Family Advisory Council represents the voice of lived experience at FNP, however, it was important to include them in the FNP team member panel rather than the FNP client panel because of their knowledge of the ongoing workings of the navigation program as well as their significant involvement in the conceptualization and development of the program. Furthermore, being a current or former client of the program is not a requirement to join the Family Advisory Council.

#### Recruitment of participants

All FNP team members (seven navigators, three managers, one Parent Advocate with Lived Experience, and 11 FAC members) were invited to participate. All FNP team members were informed about upcoming study invitations in the context of regular meeting updates and subsequently received study invitation emails. Those who responded to the study invitation emails were sent the study link, which directed them to a consent screen prior to entering the survey.

In consultation with FNP team members, a list of local public- and private-sector service providers with knowledge of navigation was generated for potential participation. Finally, Navigators were asked to contact at least two potential clients who may have an interest in participating. All potential participants were then contacted and invited to participate in the study, and those who expressed interest were sent the survey link. Potential participants were presented with a consent screen prior to entering the survey. Participants were anonymous to each other, but not to the researchers, so that the researchers could link responses to panel membership and also provide participants with copies of their own rankings at the beginning of the third phase of data collection.

### Data collection and analysis

The three phases of the Delphi Study were distributed through email and completed by participants online through SurveyMonkey®. Research ethics approval was obtained from the institutional review board. The review board was also updated and presented with the data collection tools prior to the commencement of each phase of the study. Although there are many agreed-upon methods of conducting Delphi studies, the phases outlined below were guided primarily by Hsu and Sanford [14] and Schmidt [17], who provide thorough descriptions of methods for generating lists of issues in topic areas where there is little to no existing literature and paring issues down to key factors which have reached “consensus”.

#### Phase 1

The first phase of this study took place between August 4 and September 7, 2016. Phase 1 consisted of a questionnaire that explored the three Navigation Components identified in the study purpose: 1) the key features of a successful family navigation process, 2) the features of good matches between youth/families and the services to which they are navigated, and 3) the outcomes of importance in family navigation. The questionnaire instructed participants to describe at least 6 things that came to mind when reading the specific prompts. The prompts were posed to participants in logical conceptual order, regardless of Navigation Component assignment, and were presented as follows:

Prompt 1. What do you think are the features of a successful navigation process? *(Navigation Component 1).*

Prompt 2. What do good outcomes look like for the youth? *(Navigation Component 3).*

Prompt 3. What do good outcomes look like for the whole family? *(Navigation Component 3).*

Prompt 4. What do good outcomes look like for the navigator? *(Navigation Component 3).*

Prompt 5. What do good outcomes look like for the service provider? *(Navigation Component 3).*

Prompt 6. Are there specific principles and values of navigation that lead to good outcomes? What are they? *(Navigation Component 1).*

Prompt 7. What are the factors to do with the youth that lead to a good match between the youth/family and referred service? *(Navigation Component 2).*

Prompt 8. What are the features of a service that lead to a good match between the youth/family and referred service? *(Navigation Component 2).*

Prompt 9. What are the factors to do with a family that lead to a good match between the youth/family and referred service? *(Navigation Component 2).*

During Phase 1 of data collection, participants were encouraged to submit and describe as many factors as possible to ensure that there would be sufficient information to move forward into subsequent phases [17]. Once participants completed Phase 1, the study team consolidated the statements submitted into a single list for each question. Summative qualitative content analysis [18] was utilized to group submitted statements into conceptual patterns, with consideration of the context of the submitted statements, in order to create meaningful and representative responses under each question. The list was organized such that submitted statements referring to the same concept within a prompt were collapsed into one response and so that categorically similar terms appeared together [17]. This analysis of text was completed in MaxQDA software. Once this organization of Phase 1 responses had been completed, the study proceeded to Phase 2.

#### Phase 2

The second phase of this study took place between December 5th and December 29th, 2016. The goal of Phase 2 was to reduce the responses that emerged in Phase 1 and create a list of agreed-upon factors for presentation in the subsequent phase. Participants were sent the compiled Phase 1 responses, which were presented in random order within each question and were presented in a different order to every participant to prevent any bias due to order effects. Participants were asked to select and rank the top ten most important Phase 1 responses for each prompt. By selecting from and ranking the Phase 1 responses, potential response bias was reduced in the next phase by culling listed Phase 1 responses and creating a more concise list of responses for subsequent presentation to participants [14]. Okoli & Pawloskis [19] suggest that responses selected by over 50% of respondents be carried forward to the next phase. This approach was modified to instead reflect *majority overall endorsement* of a given response since there were ten selections available to each participant for each prompt, yet the number of responses available to select from ranged from 15 to 33 within each prompt. For example, there were 32 respondents and 17 possible responses to choose from in Question 2. Since each respondent was to select and rank any ten responses, 320 selections were available to be distributed across the 17 responses. Thus, a majority would be indicated in Question 2 if any one response was selected more than 18.8 times (320/17). Participants were asked to rank their selections to make relative determinations of importance. Phase 2 responses were ordered according to the percentages of participants who had endorsed the response for presentation in Phase 3.

#### Phase 3

The third phase of this study took place between March 17 and May 5, 2017. Phase 3 was designed to assess participants’ perceptions of importance of the responses that had been retained until then. Participants were asked to review the consolidated list of Phase 2 responses and indicate their assessment of the importance of each of these responses on a 7-point likert scale (Extremely Unimportant to Extremely Important). To further support generation of consensus, participants were provided with both the overall group’s Phase 2 rankings and their own Phase 2 rankings for each response to aid them in determining whether they wished to maintain their previous assessment of a response or not. This allowed participants to confront their own agreement or disagreement with group opinion and respond accordingly [15]. The criterion cut-off for convergence within 5-point Likert-type scales is typically 80% of the subjects’ votes falling within the top two measures of the scale (i.e. a score of 4 or 5) [20]. We modified this criterion to consider consensus reached at 80% agreement within the top two scores on our 7-point Likert scale (i.e. a score of 6 or 7). This level of consensus was required across all participants, and not within each expert panel.

**Fig. 1:**
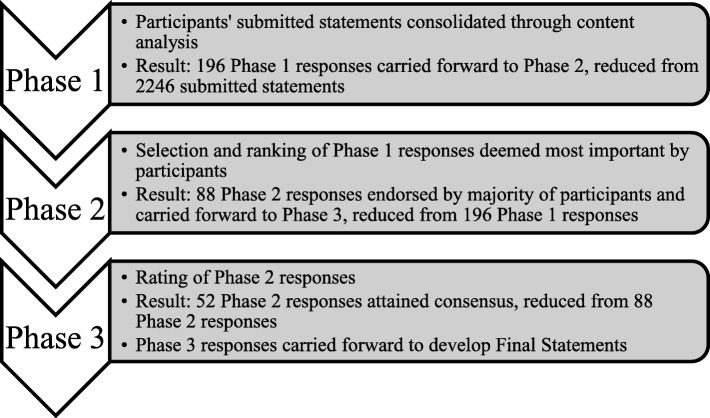
– Progression of study phases

After the three phases were completed, the final list of Phase 3 responses that were retained by participants were determined to be those responses that best represent the core features of each Navigation Component. Phase 3 responses were grouped together under their overarching Navigation Component, and redundancies were addressed. Duplicate or nearly identical responses that appeared across different Prompts within the same Navigation Component were eliminated or combined (i.e. “the navigator has expertise and broad knowledge about youth mental health and addictions…” and “knowledge and understanding of mental health and addictions system and existing services” were combined into “Expertise, knowledge, and understanding of the mental health and addictions system and existing services…”). Following this consolidation, the finalized list of statements pertaining to each Navigation Component was generated. Finally, to ascertain the level of agreement between panelists, Intraclass Correlation Coefficients (ICC) were calculated in SPSS 24 for each Navigation Component as well as for the entire finalized list of responses. See Fig. 1 for a representation of the progression of study phases.

## Results

### Phase 1

A total of 41 participants took part in Phase 1, which included 11 service providers, 14 clients, and 16 navigation team members. A total of 36 participants identified as female (87.8%) and 5 identified as male (12.2%). With respect to age, 7 participants (17.1%) were under age 35, 5 participants (12.2%) were between 35 and 45, 16 participants (39%) were between 45 and 55, and 12 participants (29.3%) were between 55 and 65. One participant declined to answer. By providing their thoughts on the prompts, 2246 total statements were generated by the 41 participants; an average of 6.1 submitted statements per prompt, per person. In some cases, as few as one participant submitted a particular statement, whereas in other instances, all participants submitted some variation of the same statement. A full list of submitted statements and the numbers of participants who provided the same statement is available in Additional file 1: Table S1. The total number of responses yielded per prompt was as follows. Prompt 1: 376. Prompt 2: 301. Prompt 3: 258. Prompt 4: 232. Prompt 5: 207. Prompt 6: 275. Prompt 7: 145. Prompt 8: 251. Prompt 9: 201. Following qualitative content analysis, these open-text responses were reduced and/or combined to reflect unique responses only. For example, submitted statements such as “offer solutions that can actually be used,” “providing realistic and relevant choices of treatment options,” “a service has been provided to them that meets their needs,” and “making me aware of other resources I might access and helping me find other appropriate resources” were grouped together under the response: “The family receives usable and practical resource options.” Some submitted statements could be included under multiple responses and as such contributed to the development of more than one Phase 1 response. For instance, “making me aware of other resources I might access and helping me find other appropriate resources” also supported the creation of the Phase 1 response: “the navigation Team removes some burden and/or stress from family by performing some of the work associated with seeking and accessing the appropriate resources.” Thus, the final numbers of responses generated per prompt in Phase 1 were: Prompt 1: 22, Prompt 2: 17, Prompt 3: 16, Prompt 4: 16, Prompt 5: 19, Prompt 6: 28, Prompt 7: 16, Prompt 8: 33, Prompt 9: 29.

### Phase 2

All Phase 1 participants were invited to take part in Phase 2; of the 41 Phase 1 participants, 32 (78.1%) continued, comprising 9 service providers, 11 clients, and 12 navigation team members. A total of 28 participants identified as female (87.5%) and 4 identified as male (12.5%). With respect to age, 5 participants (15.6%) were under age 35, 4 participants (12.5%) were between 35 and 45, 13 participants (40.6%) were between 45 and 55, and 10 participants (31.3%) were between 55 and 65. Following this round, a total of 12 responses were retained in Prompt 1, 8 responses in Prompt 2, 9 responses in Prompt 3, 9 responses in Prompt 4, 7 responses in Prompt 5, 12 responses in Prompt 6, 9 responses in Prompt 7, 10 responses in Prompt 8, and 12 responses in Prompt 9. Within Phase 1 responses that had attained majority overall endorsement (as defined above), the overall proportion of participants that had endorsed the response ranged from 42.9 to 100% (Table S1). The percentage of participants who had selected a response was used to determine the overall ranking of the response following Phase 2 (Table S1). If multiple Phase 1 responses had received same percentage endorsement, the median rank assigned by participants was the second point of comparison to determine the relative ranking of the response.

### Phase 3

All participants who had taken part in the second phase were invited to participate in Phase 3, resulting in a total of 21 participants (65.6%) of the original 41 who had taken part in all three phases. This group included 4 service providers, 10 clients, and 7 navigation team members. A total of 19 participants identified as female (90.5%) and 2 identified as male (9.5%). With respect to age, 4 participants (19%) were under age 35, 9 participants (42.9%) were between 45 and 55, and 8 participants (38.1%) were between 55 and 65. As a result of this Phase, 36 responses were removed that did not meet the 80% cutoff criterion for consensus (Table S1).

### Finalizing findings by navigation component and confirmation of agreement

The finalized list of statements for each of the three Navigation Components is presented in Table 1. The ICC for the full set of finalized statements was .84, indicating excellent agreement among panelists overall.

**Table 1:** Finalized list of statements for each of the three Navigation Components

Navigation Component	Prompts	Retained Factors	Intraclass Correlation Coefficient
1: Expert consensus on the key features of a successful navigation process	• Prompt 1: What do you think are the features of a successful navigation process? • Prompt 6: Are there specific principles and values of navigation that lead to good outcomes? What are they?	The navigator effectively determines the best fit by thoroughly understanding and considering the youth and families’ needs, collaborating with team members and service providers, and providing individualized suggestions	.741
		There is a strong relationship/rapport between the family and the Navigator (i.e. Navigator validates and reassures, is non-judgmental, empathetic, reliable, kind and the family feels a sense of trust and safety, and is comfortable contacting the navigator with questions or concerns)	
		There is strong communication between the family and the Navigation Team, and with other service providers as necessary (i.e. clear, consistent, and transparent communication that ensures clients are kept informed) (*removed Prompt 6 item “open communication”*)	
		The family receives usable and practical resource options (i.e. options are clinically sound, comfortable for family, accessible to family)	
		The family and/or youth are actively involved in creating and implementing the navigation plan (i.e. motivated and willing to engage and follow through on navigation plan)	
		The family receives the information and tools required to make informed decisions about their options	
		The Navigation Team removes some burden and/or stress from family by performing some of the work associated with seeking and accessing the appropriate resources	
		The Navigation service provides family-centered support (i.e. meeting the youth and family where they are at, working with and supporting any or all family members, trusting parents’ observations and insights, empowering families)	
		Navigators have expertise, knowledge, and understanding of mental health and addictions system and existing services (i.e. expert navigators are well-informed and keep up to date through training and time spent understanding resources) (*combined with Prompt 1 item 1 “navigator has expertise about youth mental health and the mental health and addictions system”)*	
		The Navigation service matches youth and/or families to services/resources (i.e. through expert knowledge of system and resources, conduct effective profiling, present options, and connect families to service providers in a timely fashion)	
		The Navigators show compassionate persistence (i.e. staying connected with the family, following up, continuing to seek appropriate resources if needed, flexibility in timelines for length of involvement with FNP) (*removed Prompt 1 item “continued and ongoing support”)*	
		The Navigators show empathy and compassion	
		There is flexibility in Navigation service (i.e. recognizing individuality, anticipating and responding to changes in situation, creative solutions, flexibility in contact)	
		There is an intake process that is comprehensive and accessible	
2: Expert consensus on the features of a good match between youth/families and services.	• Prompt 7: What are the factors to do with the youth that lead to a good match between the youth/family and referred service? • Prompt 8: What are the features of a service that lead to a good match between the youth/family and referred service? • Prompt 9: What are the factors to do with a family that lead to a good match between the youth/family and referred service?	The youth is willing to seek/participate in treatment (i.e. youth is willing to engage as an active participant, attends appointments, willing to learn about diagnosis and evidence-based options, open minded, etc.…)	.665
		The youth likes the program offered (i.e. youth feels connected, accepted, heard, respected; has a positive attitude toward the service; compatibility with other clients at the service, etc.…)	
		The youth recognizes/accepts there is a problem	
		Service providers have excellent interpersonal skills (i.e., intake worker is friendly and engaging; all staff are friendly, patient, passionate, caring, dedicated, kind, empathetic, compassionate, honest, etc.…)	
		Service providers are knowledgeable and up-to-date on evidence-based practice, multidisciplinary perspectives in service	
		The service is able to connect/engage with youth	
		The service is responsive (i.e. answer calls/emails in a prompt manner)	
		The family is supportive of the youth in general (i.e. respect the youth’s rights, assist the youth in accessing service, assume the youth is doing their best, etc.…)	
		The family is willing to be actively involved in the process (i.e. involvement and collaboration with the treating team, engaged in treatment process, etc.…)	
		The family is supportive of the youth’s treatment journey (i.e. respect the relationship between the youth and service, respectful of service providers, open and vulnerable to the process, etc.…)	
		The family is willing to communicate with the youth, Navigator, and service provider (i.e. cooperative and responsive, available to speak to navigator and/or service provider, able to effectively share information with navigator and/or service provider, provide feedback when needed)	
		The family is committed to change (i.e. ready, engaged, motivated to change)	
		The family is willing to follow through with recommendations	
Component 3: Expert consensus on the outcomes of importance in Navigation	• Prompt 2: What do good outcomes look like for the youth? • Prompt 3: What do good outcomes look like for the whole family? • Prompt 4: What do good outcomes look like for the navigator? • Prompt 5: What do good outcomes look like for the service provider?	The youth is connected to/engaged with supports (i.e. accessing resources that are a good fit, satisfied with resources)	.734
		The youth experiences improvement in symptoms (i.e. improvement in mental health, decreased substance use or risky behaviours, decreased stress, sense of well-being) (*removed Prompt 3 item “youth is coping better and/or improving”*)	
		The youth experiences improved daily functioning (i.e. able to leave the house, engage in activities of interest, sense of purpose) (*removed Prompt 3 item “youth is coping better and/or improving”*)	
		The youth is more motivated to take part in treatment (i.e. willing to seek treatment, contemplating change or acting to change, compliant with plan, motivated to improve)	
		The youth experiences emotional improvement (i.e. happier, self-awareness, increased maturity, better emotional regulation, etc.…)	
		The youth has more insight into their mental health and/or addictions difficulties (i.e. better understanding of their condition)	
		A less stressful home environment/family dynamic (i.e. decreased shame, isolation, guilt, blame, aggression, conflict; all members of family are safe; less stress-induced illness; members aware of their impact on the situation)	
		The family experiences improved daily functioning (i.e. all family members can manage their responsibilities, such as work, and be productive)	
		The caregivers develop improved parenting techniques (i.e. clear roles, boundaries, consequences; parents on the same page)	
		The family’s situation has improved (i.e. alleviation of stress, isolation, crisis; improvement in symptoms and behaviours; better coping; family has hope)	
		The navigator finds a suitable treatment/resource (i.e. providing a good match; providing a range of options; developing a clear plan; tracking options that did not work)	
		The family feels more supported, understood, and empowered by the Navigation Team	
		The Navigator has established a good therapeutic relationship with client (i.e. good communication, treated respectfully, family is open and transparent, youth engages/communicates with navigator)	
		The Navigation Team provides prompt and timely support (i.e. rapid intake, profiling, connection; adaptive and responsive to changing situations)	
		The service provider supports the family with a good therapeutic alliance (i.e. offer services respectfully and sensitively, listen to family, family/youth feel safe, understood, and supported, trust the service provider, are honest with the service provider, etc.…)	
		The family/youth show symptom improvement and/or make positive changes (i.e. independent, improved functioning, decreased number of hospital stays, growing confidence, desire for growth, setting long-term goals, etc.…)	
		The youth and/or family’s issues are a good match to services (i.e. good fit between youth/family’s needs and service, familiar with issues, able to provide appropriate treatment)	
		The youth and/or family commit to and engage with ongoing treatment (i.e. connect with referred provider, attend sessions, engaged in service and complete program, if applicable)	
		The client has improved tools/resources for coping/functioning (i.e. skills and strategies to be successful following completion of treatment, add: improved ability to manage stressful situations, recognizes strengths and learns from setbacks, has improved self-care skills, etc.…) (*combined with Prompt 2 item “youth has more tools and skills to use when needed”*)	

1. Consensus regarding the key features of a successful navigation process for youth with mental health and/or addictions concerns and their families. This Navigation Component was informed by Prompt 1 and 6 of the Delphi questionnaire presented to participants. The finalized list of statements that reached consensus is presented in Table 1. Successful navigation was, in general, characterized by expert information sharing and provision, open communication between providers and clients, family-centeredness, and flexibility. The ICC for Navigation Component 1 was .74, indicating good agreement.

2. Consensus regarding the features of a good “match” for youth, families, and service providers. This Navigation Component was informed by Prompts 7, 8, and 9 of the Delphi questionnaire presented to participants. The finalized list statements that reached consensus is presented in Table 1. Good matches to services were characterized by willingness and involvement on the part of the youth and families, and service providers who offer compassionate, engaging, and responsive service. The ICC for Navigation Component 2 was .66, indicating good agreement.

3. Consensus regarding the outcomes of importance to Navigators, youth with MHA, families, and service providers. This Navigation Component was informed by Prompts 2, 3, 4 and 5 of the Delphi questionnaire presented to participants. The finalized list of statements that reached consensus is presented in Table 1. Outcomes of importance included improved functioning, skills, and strategies; positive therapeutic relationships; and timeliness of service access and provision. The ICC for Navigation Component 3 was .73, indicating good agreement.

## Discussion

The factors that achieved consensus with regard to the key features of family navigation indicated that navigation needs to be a highly individualized and flexible process that engages and supports the youth and family, and involves extensive expertise for well-informed resource-matching based on a youth’s and family’s needs. The factors found to be key to an effective match in this study were a willingness to participate and a desire to change on the part of the youth and/or the family, as well as connection to service providers who could foster effective and caring connections with clients and who focused on evidence-based practice in service provision. The factors that were evident across outcomes for all groups were the desire for youth and families to experience an improvement by accessing navigation, evidenced by improved functioning, and developing skills and connecting to resources that would enable them have more effective coping strategies. All panels indicated that navigation and the services families ultimately accessed should aim to provide effective and timely service, and help families feel supported and connected to appropriate services in the mental health and addictions system.

The factors that ultimately achieved consensus were those defining features that are necessary for navigation, not simply sufficient for navigation. For example, “the family feels that accessing navigation is easy and flexible (i.e. rapid intake, availability and responsiveness of Navigator....)” is certainly something family navigation programs can do to limit service barriers encountered by families, but this is not a defining feature of navigation service specifically, or one that uniquely distinguishes navigation. Such responses, which could be considered appropriate for navigation services although not distinguishing features of navigation, tended to receive lower levels of endorsement. Responses with low levels of endorsement and low rankings were likely scrutinized more carefully by participants, particularly those who might have ranked certain responses highly in Phase 2 that were ranked lower by the group in comparison. Participants were provided with their own Phase 2 rankings and also provided with overall group rankings, to allow reassessment of their position in the context of the whole group. Such shifts toward group consensus are expected through the Delphi process, but due to group anonymity, this shift is not considered coerced [15]. Upon further scrutiny in Phase 3, participants assigned lower ratings to responses that were not unique to navigation or not defining features of navigation. Although such responses were not determined by group consensus to comprise core aspects of family navigation services, they are certainly good practice for family and youth mental health services and supports in general.

Many responses within a Navigation Component prompt were conceptually linked, in that one concept could not arise without the other. For example, “the Navigator expands their own network of resources” may occur naturally as the Navigator develops “expertise, knowledge, and understanding of mental health and addictions system and existing services.” Furthermore, a “family feels more supported” was dropped following Phase 3, but is still to be expected to arise when “there is strong communication between the family and the navigation team, and with other service providers as necessary,” a response which was retained. Forced selection and ranking persuades respondents to consider and express their opinions and make decisions around key issues of importance in order to determine group priorities [14, 17]. Thus, it is possible that even those factors that were filtered out through the Delphi process may still be important, but through the mechanisms of the navigation process may occur as a natural result of the other factors that were ultimately selected and retained in the finalized set of responses.

Of those responses that ultimately achieved consensus, it is also important to note that not every factor will be important or expected for every family in every instance. Navigation is individualized and adaptive to identified needs over the trajectory of a client’s involvement in the process [21]. Although this Delphi process has identified core factors that can help guide expectations and evaluations of navigation processes, these will not negate the individualized nature of navigation, which needs to be flexible and responsive to individual goals and needs. For instance, if a client were to reach out to a navigation service while recognizing that their youth was not ready to participate in treatment and was seeking to connect with supports that would help the caregiver improve their parenting techniques in the interim, it would not necessarily be relevant to impose expectations or unwanted treatment plans, or assess outcomes pertaining to the youth connecting to or engaging with supports, if that was not the client’s goal. Recognizing individual needs and expectations in this manner aligns with findings that emerged pertaining to the key features of navigation, such as understanding the best fit for services, providing usable and practical options, flexibility in navigation service, and family-centered support.

Low response rates are a common difficulty encountered in the Delphi method [14], and this was also noted in our panel of service providers. Overall, continued participation between phases was acceptable and yielded numbers above the recommended 15–20 participants for Delphi studies [14]. Furthermore, participation from service providers was not sufficient to make any distinctions among different kinds of providers (for example, public or private system providers). There was a higher engagement from navigation team members, which could potentially have overshadowed opinion from other groups and biased results toward those with a perspective aligned with the service model at FNP. However, the navigation team perspective was made up of management, clinicians (some of whom also had prior experience providing direct service outside of navigation), and members of the Family Advisory Council at FNP, who have the experience of caring for a youth with a mental health and/or addictions issue but may or may not have the experience of being navigated themselves. Thus, the navigation group also had membership with the experience of the other panels. Ultimately, there were no responses in any Phase of the study that were retained with sole endorsement by the navigation team, without agreement from at least one of the two other panels of service providers and clients with lived experience.

Another potential limitation is that no navigation services aside from the FNP were engaged in this study. At the time of study planning, we were unaware of other family MHA navigation teams with clinical expert Navigators. Although there is a great deal of value in peer-led services, at the time it was decided that such service models, foci, and goals of service were not necessarily aligned with the goals of this study, which focused on a model of navigation that combines clinical expert navigation and lived experience program involvement to support families and youth in accessing appropriate services. Although this may limit context, findings from this work may be transferable to other navigation service settings that are aiming to determine best practices in navigation support and tracking outcomes for families and youth with MHA concerns. Future directions may involve creating program evaluation and outcome evaluation tools based on these responses and validating them with larger groups, broader navigation program representation, and broader stakeholder representation (e.g., those with expertise in family engagement, family empowerment, peer support, etc.…). Other navigation services will then be able to select from these statements and craft process and outcome measures for their own use, serving to develop the literature base in this growing area of mental health and addictions services. Future studies with a qualitative perspective are also worthy of consideration, to glean rich perspectives from the voice of lived experience of youth and families and service providers and for in-depth understanding of how navigation programs support youth and families in achieving outcomes that are meaningful to them.

## Conclusions

As navigation services are becoming increasingly available and are gaining recognition as a viable way to support access to and coordination of care for individuals with MHA concerns and their loved ones, it is essential that there be shared understandings of family navigation; what core features are present in family navigation services, expectations of outcomes for those involved in family navigation services, and how successful matches are made through the navigation process. Through this Delphi process, we have established statements that can provide guidance for navigation service delivery and that can be explored to support outcome evaluation in family navigation services. This study may be an important step in creating a shared dialogue for established and emerging navigation services, the community service providers with which they interact, and most importantly, youth with MHA issues and their families who are seeking support.

## Additional file

Additional file 1: **Table S1.** – Results from all Phases (DOCX 52 kb)

## Abbreviations

FNP: Family Navigation Project
MHA: mental health and/or additions
