# Supplementary material for: Identifying the key features and outcomes of family navigation services for mental health and/or addictions concerns: a Delphi study
Source: BMC Health Serv Res. 2019 Feb 28;19:137. doi: 10.1186/s12913-019-3968-6 (PMC6393957; doi:10.1186/s12913-019-3968-6)
Supplement: Supplementary file 1 — Table S1. – Results from all Phases (DOCX 52 kb) [file 12913_2019_3968_MOESM1_ESM.docx]

**Table S1** Results from all Phases

|  | | **Phase 1** | **Phase 2** | | | | **Phase 3** | |
| --- | --- | --- | --- | --- | --- | --- | --- | --- |
|  | | **Listed (n)** | **Item in top 10 (%)** | **Mean Rank** | **Median Rank** | **Phase Rank** | **Rated very or extremely important (%)** | **Final Result** |
| **Q1: Features of a successful navigation process** | | | | | | | | |
| The navigator has expertise and broad knowledge about youth mental health and addictions and the mental health and addictions system | | 48 | 68.8 | 6.2 | 7 | 3 | 18 (90) | Retain |
| The navigator effectively determines the best fit by thoroughly understanding and considering the youth and families’ needs, collaborating with team members and service providers, and providing individualized suggestions | | 47 | 71.9 | 4.2 | 4 | 2 | 20 (100) | Retain |
| There is a strong relationship/ rapport between the family and the Navigator (i.e. Navigator validates and reassures, is non-judgmental, empathetic, reliable, kind and the family feels a sense of trust and safety, and is comfortable contacting the navigator with questions or concerns) | | 39 | 56.3 | 4.7 | 5.5 | 9 | 16 (80) | Retain |
| There is strong communication between the family and the Navigation Team, and with other service providers as necessary (i.e. clear, consistent, and transparent communication that ensures clients are kept informed) | | 37 | 62.5 | 6.5 | 7 | 8 | 16 (80) | Retain |
| The entire family is supported and/or guided | | 36 | 25.0 |  |  | Drop |  |  |
| There is continued and ongoing availability of support from the Navigation Team (i.e. regular check-in and/or continued involvement until no longer needed) | | 28 | 53.1 | 5.6 | 6 | 12 | 16 (80) | Retain |
| The family receives usable and practical resource options (i.e. options are clinically sound, comfortable for family, accessible to family) | | 24 | 75.0 | 5.2 | 5 | 1 | 20 (100) | Retain |
| The family feels listened to and understood | | 14 | 37.5 |  |  | Drop |  |  |
| The family gains knowledge about the youth’s concerns and the mental health and addictions system and feels empowered in dealing with the system | | 12 | 34.4 |  |  | Drop |  |  |
| The family feels that accessing FNP is easy and flexible (i.e. rapid intake, availability and responsiveness of navigator, flexibility in method of service) | | 12 | 62.5 | 5.2 | 4 | 5 | 15 (75) | Drop |
| The family and/or youth are actively involved in creating and implementing the navigation plan (i.e. motivated and willing to engage and follow through on navigation plan) | | 12 | 62.5 | 5.8 | 6 | 7 | 18 (90) | Retain |
| Family feels that their needs and goals have been met by FNP (i.e. family functioning has improved, sense that they have moved forward) | | 11 | 34.4 |  |  | Drop |  |  |
| The family receives the information and tools required to make informed decisions about their options | | 10 | 56.3 | 6.1 | 6 | 10 | 17 (85) | Retain |
| Family and youth report that their goals and navigation plan are clear and individualized | | 9 | 18.8 |  |  |  |  | Drop |
| The Navigation Team promptly connects the youth and family to appropriate resources | | 8 | 62.5 | 5.2 | 5 | 6 | 17 (85) | Retain |
| The Navigation Team removes some burden and/or stress from family by performing some of the work associated with seeking and accessing the appropriate resources | | 7 | 65.6 | 5.3 | 5 | 4 | 18 (90) | Retain |
| The Navigation Team has respect for confidentiality and appropriate documentation of consent | | 7 | 18.8 |  |  | Drop |  |  |
| The family believes they have someone who will advocate on their behalf | | 6 | 40.6 |  |  | Drop |  |  |
| There is an intake process that is easy to understand and sensitive (i.e. streamlined, information sharing with consent, ensuring clients do not constantly have to repeat information already provided) | | 4 | 53.1 | 4.8 | 5 | 11 | 14 (70) | Drop |
| Lived experience is integrated into the model (i.e. lived experience informs service delivery and/or families have access to peer support) | | 3 | 40.6 |  |  | Drop |  |  |
| The family has access to financial resources at the time they contact FNP | | 1 | 0 |  |  | Drop |  |  |
| Navigation service is trauma-informed | | 1 | 0 |  |  | Drop |  |  |
| **Q2: Good outcomes for the youth** | | | | | | | | |
| Youth is connected to/engaged with supports (i.e. accessing resources that are a good fit, satisfied with resources) | | 64 | 81.3 | 4.4 | 4 | 2 | 18 (90) | Retain |
| Youth has more tools and skills to use when needed (i.e. has improved ability to manage stressful situations, recognizes strengths and learns from setbacks, has improved self-care skills, etc…) | | 29 | 75.0 | 4.7 | 5 | 3 | 17 (85) | Retain |
| Youth experiences improvement in symptoms (i.e. improvement in mental health, decreased substance use or risky behaviours, decreased stress, sense of well-being) | | 29 | 71.9 | 3.6 | 3 | 4 | 18 (90) | Retain |
| Youth has improved engagement with school, work, or social activities | | 25 | 46.9 |  |  | Drop |  |  |
| Youth has good rapport with care providers (i.e. feels understood, respected, and heard, willing to open up) | | 23 | 46.9 |  |  | Drop |  |  |
| Youth sets individual goals and works toward them | | 19 | 34.4 |  |  | Drop |  |  |
| Youth experiences improved daily functioning (i.e. able to leave the house, engage in activities of interest, sense of purpose) | | 18 | 81.3 | 4.4 | 4 | 1 | 19 (95) | Retain |
| Youth experiences improved relationships and communication with family | | 17 | 68.8 | 5.7 | 6 | 7 | 12 (60) | Drop |
| Youth is more motivated to take part in treatment (i.e. willing to seek treatment, contemplating change or acting to change, compliant with plan, motivated to improve) | | 16 | 59.4 | 4.8 | 3.5 | 8 | 16 (80) | Retain |
| Youth experiences emotional improvement (i.e. happier, self-awareness, increased maturity, better emotional regulation, etc…) | | 11 | 68.8 | 4.7 | 4.5 | 6 | 18 (90) | Retain |
| Youth has improved confidence and self-esteem | | 11 | 46.9 |  |  | Drop |  |  |
| Youth has more insight into their mental health and/or addictions difficulties (i.e. better understanding of their condition) | | 10 | 71.9 | 6.5 | 6.5 | 5 | 17 (85) | Retain |
| Youth feels more empowered in his/her care | | 8 | 46.9 |  |  | Drop |  |  |
| Youth feels more in control of his/her own life | | 6 | 53.1 |  |  | Drop |  |  |
| Family is receiving/willing to receive support | | 4 | 56.3 |  |  | Drop |  |  |
| Youth feels like he/she is less alone | | 4 | 53.1 |  |  | Drop |  |  |
| Youth feels personal growth (i.e. has developed independence, resilience, believes in self, etc…) | | 7 | 37.5 |  |  | Drop |  |  |
| **Q3: Good outcomes for the whole family** | | | | | | | | |
| There is a less stressful home environment/family dynamic (i.e. decreased shame, isolation, guilt, blame, aggression, conflict; all members of family are safe; less stress-induced illness; members aware of their impact on the situation) | | 36 | 87.1 | 4.5 | 5 | 1 | 16 (88.9) | Retain |
| Whole family is accessing support (i.e. accessing resources that are a good fit, working toward positive change) | | 29 | 41.9 |  |  | Drop |  |  |
| Family feels more supported (i.e. feels heard, have someone to reach out to, feel as though have access to ongoing support) | | 25 | 64.5 | 5.8 | 6 | 9 | 14 (77.8) | Drop |
| Family experiences improvement in communication | | 25 | 58.1 |  |  | Drop |  |  |
| Family develops increased knowledge of youth mental health and/or addictions and the mental health/addictions system | | 21 | 61.3 | 5.6 | 6 | 6 | 12 (66.7) | Drop |
| Family experiences improvement in relationships (including mutual respect, improved boundaries, and secure attachment relationships) | | 20 | 93.5 | 5.2 | 6 | 6 | 14 (77.8) | Drop |
| Family experiences improved problem-solving skills (i.e. better coping, better management of crises, work together to solve problems) | | 16 | 74.2 | 4.8 | 5 | 6 | 13 (72.2) | Drop |
| Family is more cohesive (i.e. all family members contribute, sense of family balance, cohesiveness) | | 15 | 48.4 |  |  | Drop |  |  |
| All family members more involved in supporting the youth | | 13 | 29.0 |  |  | Drop |  |  |
| Youth is coping better and/or improving | | 11 | 74.2 | 2.3 | 1 | 1 | 16 (88.9) | Retain |
| Family experiences improved daily functioning (i.e. all family members can manage their responsibilities, such as work, and be productive) | | 10 | 83.9 | 5.6 | 4.5 | 4 | 17 (94.4) | Retain |
| Family issues are improving (i.e. sense of achievement, on a clear path forward) | | 10 | 58.1 |  |  | Drop |  |  |
| Caregivers develop improved parenting techniques (i.e. clear roles, boundaries, consequences; parents on the same page) | | 8 | 67.7 | 5.8 | 6 | 7 | 15 (83.3) | Retain |
| Family feels more hopeful | | 7 | 77.4 | 5.9 | 6.5 | 7 | 13 (72.2) | Drop |
| Family feels more empowered to advocate for youth | | 6 | 48.4 |  |  | Drop |  |  |
| Family feels happier | | 6 | 32.3 |  |  | Drop |  |  |
| **Q4: Good outcomes for the navigator/navigation team** | | | | | | | | |
| The family’s situation has improved (i.e. alleviation of stress, isolation, crisis; improvement in symptoms and behaviours; better coping; family has hope) | | 47 | 93.3 | 4.7 | 5 | 7 | 17 (94.5) | Retain |
| Navigator finds a suitable treatment/resource (i.e. providing a good match; providing a range of options; developing a clear plan; tracking options that did not work) | | 33 | 90.0 | 3.9 | 3 | 3 | 17 (94.5) | Retain |
| Navigator expands their own network of resources (i.e. maintaining a network of professionals, gaining knowledge and experience, having a positive impact on the system by directing families to appropriate resources) | | 22 | 70.0 | 7.6 | 8 | 10 | 12 (66.7) | Drop |
| Family feels more supported, understood, and empowered by the Navigation Team | | 21 | 80.0 | 5.0 | 5 | 2 | 15 (83.3) | Retain |
| Navigation Team receives positive feedback about the match/resource (i.e. from families and/or service providers) | | 17 | 60.0 | 7.5 | 8 | 9 | 10 (55.6) | Drop |
| Navigator has established a good therapeutic relationship with client (i.e. good communication, treated respectfully, family is open and transparent, youth engages/communicates with navigator) | | 17 | 93.3 | 3.8 | 3 | 1 | 15 (83.3) | Retain |
| Navigation Team provides prompt and timely support (i.e. rapid intake, profiling, connection; adaptive and responsive to changing situations) | | 14 | 80.0 | 4.3 | 3.5 | 1 | 17 (94.5) | Retain |
| Family pursues options/connects with resources | | 12 | 80.0 | 4.8 | 4.5 | 6 | 11 (61.1) | Drop |
| Family re-connects as needed/remains engaged | | 11 | 40.0 |  |  |  |  | Drop |
| Navigators feel part of an effective and collaborative team (i.e. collaborate with team members to develop options, do not feel overwhelmed, manageable caseload) | | 9 | 86.7 | 5.8 | 6 | 9 | 13 (72.2) | Drop |
| Navigator is satisfied with assistance provided to the family (i.e. confident they have found good options for the family) | | 7 | 53.3 |  |  | Drop |  |  |
| Youth demonstrates willingness to participate in options | | 7 | 56.7 |  |  | Drop |  |  |
| Family needs less and less support from the navigator | | 7 | 63.3 |  |  | Drop |  |  |
| Family expresses gratitude toward the navigator | |  | 30.0 |  |  | Drop |  |  |
| Navigator receives increased number of new client referrals from satisfied clients | | 5 | 16.7 |  |  | Drop |  |  |
| Number of new clients increases year to year | | 2 | 0 |  |  | Drop |  |  |
| Navigator improves their own knowledge base in cultural awareness (i.e. more accessible to culturally diverse clients) | | 1 | 6. 7 |  |  | Drop |  |  |
| **Q5: Good outcomes for the service provider** | | | | | | | | |
| Service provider supports the family with a good therapeutic alliance (i.e. offer services respectfully and sensitively, listen to family, family/youth feel safe, understood, and supported, trust the service provider, are honest with the service provider, | | 24 | 86.7 | 4.3 | 4 | 3 | 18 (100) | Retain |
| Service provider collaborates with navigator on shared cases (i.e. warm handoff from FNP to service provider, mutual understanding of roles, FNP follows up, good relationship with navigator) | | 23 | 73.3 | 5.9 | 6.5 | 3 | 13 (72.2) | Drop |
| Family/youth show symptom improvement and/or make positive changes (i.e. independent, improved functioning, decreased number of hospital stays, growing confidence, desire for growth, setting long-term goals, etc…) | | 22 | 93.3 | 4.4 | 5 | 5 | 17 (94.4) | Retain |
| Youth and/or family’s issues are a good match to services (i.e. good fit between youth/family’s needs and service, familiar with issues, able to provide appropriate treatment) | | 21 | 86.7 | 3.7 | 4 | 4 | 18 (100) | Retain |
| Youth and/or family commit to and engage with ongoing treatment (i.e. connect with referred provider, attend sessions, engaged in service and complete program, if applicable) | | 21 | 90.0 | 5.1 | 5 | 2 | 15 (83.3) | Retain |
| The service is more accessible to families (i.e. able to provide service in a prompt manner, simplified intake process) | | 15 | 60.0 |  |  | Drop |  |  |
| Service provider receives clients who are a better fit (i.e. appropriate referrals, navigators profiled well, etc…) | | 15 | 60.0 |  |  | Drop |  |  |
| Service provider is able to provide options for other services or after-care when needed | | 14 | 60.0 |  |  | Drop |  |  |
| Service provider receives positive feedback and/or outcome evaluations of treatment provided (i.e. from youth/family, navigator, etc…) | | 9 | 43.3 |  |  | Drop |  |  |
| Client has improved tools/resources for coping/functioning (i.e. skills and strategies to be successful following completion of treatment) | | 8 | 76.7 | 5.3 | 5 | 9 | 16 (88.9) | Retain |
| Service provider's reputation grows (i.e. receive more clients, clients recommend service to others, increased funding for programming, etc…) | | 7 | 26.7 |  |  | Drop |  |  |
| Service provider provides knowledge and education regarding mental health and/or addictions issues to youth and/or families | | 5 | 43.3 |  |  | Drop |  |  |
| Service provider, youth/family, and Navigator maintain strong communication | | 5 | 50.0 |  |  | Drop |  |  |
| Service provider develops increased awareness of resources in the community (including the Family Navigation Project) | | 4 | 50.0 |  |  | Drop |  |  |
| Youth and/or family achieves goals | | 4 | 86.7 | 4.4 | 3 | 1 | 12 (66.7) | Drop |
| Youth and/or family are open to reconnect as needed | | 3 | 3.3 |  |  | Drop |  |  |
| Service provider helps ensure realistic expectations are defined by all parties | | 3 | 6.7 |  |  | Drop |  |  |
| Family shows patience with progress | | 2 | 3.33 |  |  | Drop |  |  |
| Service providers are not overwhelmed | | 2 | 0 |  |  | Drop |  |  |
| **Q6: Important principles and values of navigation** | | | | | | | | |
| Family-centered support (i.e. meeting the youth and family where they are at, working with and supporting any or all family members, trusting parents’ observations and insights, empowering families) | | 38 | 62.1 | 4.3 | 3.5 | 1 | 15 (83.3) | Retain |
| Knowledge and understanding of mental health and addictions system and existing services (i.e. expert navigators are well-informed and keep up to date through training and time spent understanding resources) | | 24 | 75.9 | 4.1 | 3 | 3 | 18 (100) | Retain |
| Rapport with families and youth (i.e. building a positive relationship with respect, trust, and reliability for effective collaboration) | | 16 | 34.5 |  |  | Drop |  |  |
| Matching youth and/or families to services/resources (i.e. through expert knowledge of system and resources, conduct effective profiling, present options, and connect families to service providers in a timely fashion) | | 16 | 69.0 | 3.7 | 3 | 1 | 18 (100) | Retain |
| Compassionate persistence (i.e. staying connected with the family, following up, continuing to seek appropriate resources if needed, flexibility in timelines for length of involvement with Family Navigation Project) | | 16 | 62.1 | 5.2 | 6 | 6 | 15 (83.3) | Retain |
| Open communication (i.e. keeping clients informed, being responsive, maintaining transparency in communication) | | 15 | 44.8 | 6.8 | 7 | 8 | 15 (83.3) | Retain |
| Empathy and compassion | | 14 | 48.3 | 5.3 | 6.5 | 1 | 16 (88.9) | Retain |
| Team process of support and collaboration (i.e. team consultation at FNP, collaboration and knowledge-sharing, navigator debrief and self-care) | | 14 | 41.4 | 7.3 | 7.5 | 6 | 14 (77.8) | Drop |
| Focus on client preferences (i.e. strengths-based approach, understanding of clients’ needs while respecting their own expertise regarding their needs) | | 14 | 20.7 |  |  | Drop |  |  |
| Flexibility in navigation service (i.e. recognizing individuality, anticipating and responding to changes in situation, creative solutions, flexibility in contact) | | 13 | 51.7 | 5.3 | 4 | 3 | 16 (88.9) | Retain |
| Intake process that is comprehensive and accessible | | 10 | 41.4 | 4.7 | 4.5 | 4 | 16 (88.9) | Retain |
| Honesty with families (i.e. setting realistic expectations, providing an objective perspective) | | 10 | 34.5 |  |  | Drop |  |  |
| Non-judgmental interactions with families | | 9 | 31.0 |  |  | Drop |  |  |
| Navigation staff have strong listening skills | | 9 | 10.3 |  |  | Drop |  |  |
| Rapport and collaboration with other service providers | | 8 | 24.1 |  |  | Drop |  |  |
| The Navigation Team has good interpersonal skills (i.e. good judgment, kindness, intuition, integrity, humour, etc…) | | 7 | 37.9 |  |  | Drop |  |  |
| The Navigation Team ensures confidentiality | | 6 | 24.1 |  |  | Drop |  |  |
| Instilling hope and being optimistic | | 5 | 34.5 |  |  | Drop |  |  |
| Engagement (i.e. getting in the boat with families) | | 4 | 51.7 | 5.0 | 4 | 4 | 13 (72.2) | Drop |
| Negotiating challenging situations for families | | 4 | 44.8 | 6.5 | 7 | 4 | 14 (77.8) | Drop |
| A model that brings together clinical and lived experience | | 4 | 44.8 | 6.3 | 6 | 5 | 14 (77.8) | Drop |
| Advocacy for youth and/or families | | 4 | 20.7 |  |  | Drop |  |  |
| Establish and support a circle of care | | 3 | 24.1 |  |  | Drop |  |  |
| Patience with the process | | 3 | 13.8 |  |  | Drop |  |  |
| Regularly obtaining client and staff input (i.e. regular measurement and evaluation) | | 3 | 10.3 |  |  | Drop |  |  |
| Community engagement | | 2 | 6.9 |  |  | Drop |  |  |
| Relationship-based care | | 2 | 24.1 |  |  | Drop |  |  |
| Cultural sensitivity | | 2 | 10.3 |  |  | Drop |  |  |
| **Q7: Factors related to the youth that lead to a good match between youth/family and referred service** | | | | | | | | |
| Youth is willing to seek/participate in treatment (i.e. youth is willing to engage as an active participant, attends appointments, willing to learn about diagnosis and evidence-based options, open minded, etc…) | | 33 | 93.1 | 3.6 | 3 | 1 | 15 (83.3) | Retain |
| Youth expresses openness with respect to perspectives, needs, goals, and preferences for treatment | | 18 | 82.8 | 5.5 | 7 | 7 | 8 (44.5) | Drop |
| Youth is motivated to improve their symptoms or functioning (i.e. desire to get better, willing to work for change) | | 16 | 100 | 3.3 | 3 | 4 | 12 (66.7) | Drop |
| Youth likes the program offered (i.e. youth feels connected, accepted, heard, respected; has a positive attitude toward the service; compatibility with other clients at the service, etc…) | | 15 | 96.6 | 4.9 | 4 | 4 | 15 (83.3) | Retain |
| Youth is involved with his/her family (i.e. youth actively interacts with family, family is supportive, etc…) | | 10 | 86.2 | 6.8 | 7 | 6 | 7 (38.9) | Drop |
| The nature and severity of the youth’s mental health and/or addictions issues (i.e. stability and/or severity of symptoms, number of previous attempts for treatment, length of time the youth has been struggling, etc…) | | 9 | 72.4 | 5.7 | 5 | 7 | 9 (50) | Drop |
| Youth has pre-existing supports in place (i.e. supportive friends, supportive school or workplace, etc…) | | 7 | 41.4 |  |  | Drop |  |  |
| Youth recognizes/accepts there is a problem | | 7 | 86.2 | 3.5 | 2.5 | 1 | 15 (83.3) | Retain |
| Youth is willing to consult with navigator | | 4 | 69.0 | 6.8 | 7 | 6 | 6 (33.3) | Drop |
| Youth is comfortable with suggestions provided | | 3 | 86.2 | 6.1 | 6 | 6 | 14 (77.8) | Drop |
| Youth has access to financial resources (i.e. themselves or through family/other supports) | | 3 | 24.1 |  |  | Drop |  |  |
| Youth is patient (i.e. with accessing service, developing rapport, seeing results of treatment, etc…) | | 3 | 37.9 |  |  | Drop |  |  |
| Youth has flexibility to use different modes of support (i.e. online, text, face to face, etc…) | | 2 | 62.1 |  |  | Drop |  |  |
| Youth is hopeful and/or optimistic | | 2 | 31.0 |  |  | Drop |  |  |
| Youth is not actively symptomatic and/or abusing substances | | 1 | 31.0 |  |  | Drop |  |  |
| **Q8: Features of a referred service that lead to a good match between the youth/family and referred services** | | | | | | | | |
| Service is accessible (i.e. ease of contact and intake, geographically accessible, no/short wait list, scheduling availability) | | 29 | 42.9 | 4 | 4 | 8 | 14 (77.8) | Drop |
| Service has flexibility in delivery | | 18 | 28.6 |  |  | Drop |  |  |
| Service providers have excellent interpersonal skills (i.e. , intake worker is friendly and engaging; all staff are friendly, patient, passionate, caring, dedicated, kind, empathetic, compassionate, honest, etc…) | | 18 | 42.9 | 5.8 | 6.5 | 10 | 17 (94.5) | Retain |
| Service is clear about niche/client issues served best (i.e. clear eligibility criteria, experience with issues at hand) | | 18 | 25.0 |  |  | Drop |  |  |
| Service is willing to work with the family and/or to collaborate with other services working with the family | | 18 | 46.4 | 4.9 | 5 | 5 | 14 (77.8) | Drop |
| Service is responsive to individual needs (i.e. client-centered, treat each client as an individual, respect client preferences, meet clients where they are at, etc…) | | 15 | 39.3 |  |  | Drop |  |  |
| Service helps create clear expectations (i.e. providing the treatment they say they will, creating clear pathways with discussion of timelines, providing meaningful information about what to expect, clear about their role, no surprises) | | 11 | 39.3 |  |  | Drop |  |  |
| Service considers the whole family (i.e. bring families together, support youth and family, treat families with respect, etc…) | | 11 | 46.4 | 6.5 | 7 | 7 | 12 (66.7) | Drop |
| Service offers a variety of programming (i.e. creative programming, drop-in services, support groups for families and youth, etc…) | | 10 | 28.6 |  |  | Drop |  |  |
| Service providers are knowledgeable and up-to-date on evidence-based practice, multidisciplinary perspectives in service | | 9 | 50.0 | 5.4 | 6 | 4 | 18 (100) | Retain |
| Service is able to connect/engage with youth | | 8 | 50.0 | 4.2 | 4 | 3 | 16 (88.9) | Retain |
| Service is affordable (i.e. financially accessible, offer sliding scale, offer TTC reimbursement, etc…) | | 8 | 46.4 | 5.7 | 6 | 6 | 14 (77.8) | Drop |
| Service is action-oriented (i.e. proactive/responsive prior to crisis, address critical needs and provide ongoing support, implement strategies for immediate relief and positive long-term coping) | | 7 | 42.9 | 5.7 | 7 | 9 | 14 (77.8) | Drop |
| Service fosters open communication | | 7 | 14.3 |  |  | Drop |  |  |
| Service is willing to work with navigator on the case | | 7 | 32.1 |  |  | Drop |  |  |
| Service ensures a focus on therapeutic alliance from onset (i.e. take time and effort to build a relationship, good boundaries) | | 6 | 39.3 |  |  | Drop |  |  |
| Service is open to feedback (e.g. regarding progress, service in general, etc…) | | 5 | 21.4 |  |  | Drop |  |  |
| Service considers overall wellness (i.e. focus on personal growth, look beyond immediate needs, recognize there are many paths to recovery) | | 5 | 53.6 | 4.3 | 4 | 2 | 13 (72.2) | Drop |
| Service has lived experience perspective within the team | | 5 | 25.0 |  |  | Drop |  |  |
| Service provides support during transitions in care | | 5 | 25.0 |  |  | Drop |  |  |
| Service is responsive (i.e. answer calls/emails in a prompt manner) | | 5 | 60.7 | 4.5 | 4 | 1 | 16 (88.9) | Retain |
| Service has clear processes for intake | | 3 | 25.0 |  |  | Drop |  |  |
| Service is non-judgmental | | 3 | 21.4 |  |  | Drop |  |  |
| Service encourages youth to take responsibility for own well-being | | 3 | 10.7 |  |  | Drop |  |  |
| Service provides a safe and inclusive environment | | 3 | 39.3 |  |  | Drop |  |  |
| Service has the capacity to support different ethno-cultural concerns | | 2 | 7.1 |  |  | Drop |  |  |
| Service conducts progress evaluations and/or provides discharge summaries | | 2 | 7.1 |  |  | Drop |  |  |
| Service helps manage future aftercare and expectations | | 2 | 25.0 |  |  | Drop |  |  |
| Service has a good reputation | | 2 | 3.6 |  |  | Drop |  |  |
| Service is easy on clients who miss appointments | | 2 | 14.3 |  |  | Drop |  |  |
| Service is youth-focused/youth-centered | | 2 | 39.3 |  |  | Drop |  |  |
| Service has capacity to support different languages | | 1 | 0 |  |  | Drop |  |  |
| Service has capacity to support LGBTQ-specific concerns | | 1 | 7.1 |  |  | Drop |  |  |
| **Q9: Factors related to the family that lead to a good match between the youth/family and referred service** | | | | | | | | |
| Family is supportive of the youth in general (i.e. respect the youth’s rights, assist the youth in accessing service, assume the youth is doing their best, etc…) | 17 | 57.1 | 5 | 4.5 | 6 | 15 (83.3) | Retain |  |
| Family is willing to be actively involved in the process (i.e. involvement and collaboration with the treating team, engaged in treatment process, etc…) | 16 | 67.9 | 5.5 | 5 | 2 | 17 (94.5) | Retain |  |
| Family is supportive of the youth's treatment journey (i.e. respect the relationship between the youth and service, respectful of service providers, open and vulnerable to the process, etc…) | 14 | 60.7 | 3.9 | 3 | 3 | 18 (100) | Retain |  |
| Family is willing to communicate with the youth, Navigator, and service provider (i.e. cooperative and responsive, available to speak to navigator and/or service provider, able to effectively share information with navigator and/or service provider, provide feedback when needed | 13 | 71.4 | 4.9 | 4 | 1 | 17 (94.4) | Retain |  |
| Family is committed to change (i.e. ready, engaged, motivated to change) | 11 | 50.0 | 4.9 | 5 | 8 | 16 (88.9) | Retain |  |
| Family is interested in doing the work (i.e. open to practice strategies, understand they are responsible for their progress) | 10 | 50.0 | 5.3 | 5 | 10 | 14 (77.8) | Drop |  |
| Family is patient with the process and recognizes progress made | 10 | 28.6 |  |  | Drop |  |  |  |
| Family is willing to engage with service provider for education and support (i.e. willing to learn) | 10 | 60.7 | 6.2 | 7 | 5 | 12 (66.7) | Drop |  |
| Family is open to suggestions (i.e. willing to listen to professional advice, flexible about what they’re looking for and receptive to options, etc…) | 9 | 60.7 | 4.6 | 4 | 4 | 13 (72.2) | Drop |  |
| Family is willing to access support/participate in treatment for themselves | 9 | 42.9 | 5.6 | 6.5 | 12 | 12 (66.7) | Drop |  |
| Family is open and honest about what is happening, goals, etc… | 8 | 53.6 | 5.3 | 5 | 7 | 14 (77.8) | Drop |  |
| Family understands the youth’s mental health and/or addictions issues or willing to learn about them | 8 | 46.4 | 5.7 |  | 11 | 14 (77.8) | Drop |  |
| Family situation is stable (i.e. caregivers on same page, family able to work as a team, stability of home environment, family’s own mental health challenges and needs, etc…) | 7 | 14.3 |  |  | Drop |  |  |  |
| Family recognizes the scope and ownership of problem (i.e. awareness of situation, willing to take accountability) | 7 | 28.6 |  |  | Drop |  |  |  |
| Family has access to financial resources | 7 | 17.9 |  |  | Drop |  |  |  |
| Family has clear expectations and goals for recovery and timelines | 6 | 10.7 |  |  | Drop |  |  |  |
| Family understands the treatment process (i.e. services provided, the value of therapy, etc…) | 5 | 14.3 |  |  | Drop |  |  |  |
| Family is willing to improve family communication | 5 | 25.0 |  |  | Drop |  |  |  |
| Family feels supported and listened to by service |  | 28.6 |  |  | Drop |  |  |  |
| Family understands youth mental health or addictions and/or is willing to learn | 4 | 25.0 |  |  | Drop |  |  |  |
| The whole family is involved | 4 | 10.7 |  |  | Drop |  |  |  |
| Family is willing to follow through with recommendations | 4 | 50.0 | 5.0 | 5 | 9 | 16 (88.9) | Retain |  |
| Family is willing to put aside personal differences | 3 | 25.0 |  |  | Drop |  |  |  |
| Family is able to self-advocate | 2 | 3.57 |  |  | Drop |  |  |  |
| Family is determined to find the right services | 2 | 25.0 |  |  | Drop |  |  |  |
| Family is hopeful and/or optimistic | 2 | 28.6 |  |  | Drop |  |  |  |
| Family is willing to reach out for help | 2 | 14.3 |  |  | Drop |  |  |  |
| The youth is able to engage with the family | 2 | 28.6 |  |  | Drop |  |  |  |
